# Potently neutralizing human mAbs against the zoonotic pararubulavirus Sosuga virus

**DOI:** 10.1172/jci.insight.166811

**Published:** 2023-04-24

**Authors:** Helen M. Parrington, Nurgun Kose, Erica Armstrong, Laura Handal, Summer Diaz, Joseph Reidy, Jinhui Dong, Guillaume B.E. Stewart-Jones, Punya Shrivastava-Ranjan, Shilpi Jain, César G. Albariño, Robert H. Carnahan, James E. Crowe

**Affiliations:** 1 Department of Pathology, Microbiology and Immunology,; 2 Vanderbilt Vaccine Center, and Department of Pediatrics, Vanderbilt University Medical Center, Nashville, Tennessee, USA.; 3 Vaccine Research Center, NIH, Bethesda, Maryland, USA.; 4 Viral Special Pathogens Branch, Division of High Consequence Pathogens and Pathology, CDC, Atlanta, Georgia, USA.

**Keywords:** Immunology, Infectious disease, Adaptive immunity, Immunoglobulins

## Abstract

Sosuga virus (SOSV) is a recently discovered paramyxovirus with a single known human case of disease. There has been little laboratory research on SOSV pathogenesis or immunity, and no approved therapeutics or vaccines are available. Here, we report the discovery of human mAbs from the circulating memory B cells of the only known human case and survivor of SOSV infection. We isolated 6 mAbs recognizing the functional attachment protein hemagglutinin-neuraminidase (HN) and 18 mAbs against the fusion (F) protein. The anti-HN mAbs all targeted the globular head of the HN protein and could be organized into 4 competition-binding groups that exhibited epitope diversity. The anti-F mAbs can be divided into pre- or postfusion conformation-specific categories and further into 8 competition-binding groups. The only Ab in the panel that did not display neutralization activity was the single postfusion-specific anti-F mAb. Most of the anti-HN mAbs were more potently neutralizing than the anti-F mAbs, with mAbs in 1 of the HN competition-binding groups possessing ultrapotent (<1 ng/mL) half-maximal inhibitory virus neutralization values. These findings provide insight into the molecular basis for human Ab recognition of paramyxovirus surface proteins and the mechanisms of SOSV neutralization.

## Graphical abstract

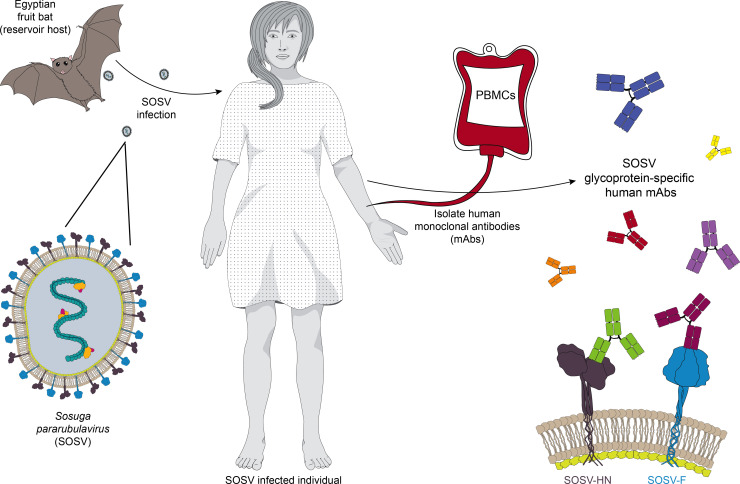

## Introduction

Paramyxoviruses are enveloped, single-stranded, negative-sense RNA viruses with a broad range of hosts, including mammals, birds, and fish (1). Paramyxoviruses have a long evolutionary history replicating in various bat species (2, 3), and bats have been the source of several zoonotic paramyxovirus spillovers into human populations (4–7). Therefore, understanding human immune responses against zoonotic and endemic paramyxoviruses is important for increasing our knowledge of the fundamental basis of immunity to these viruses and for informing the development of candidate therapeutic Abs or vaccines.

Sosuga virus (SOSV) is a recently discovered zoonotic paramyxovirus that caused a near-fatal, acute febrile disease in a wildlife researcher who had been conducting surveillance studies in South Sudan and Uganda (8, 9). The source of the infection was traced back to Egyptian rousette (*Rousettus aegyptiacus*) bats, which likely serve as an animal reservoir of this virus (9). As SOSV only recently emerged, much remains unknown about this virus, including its potential to cause human epidemics. The severe disease in the index case and the high mortality rates of other bat-borne paramyxoviruses, such as Nipah virus (10, 11), suggest that SOSV may have the potential to cause epidemics of life-threatening disease.

SOSV is classified as a paramyxovirus in the *Rubulavirinae* subfamily, and the viral genome encodes the attachment protein hemagglutinin-neuraminidase (HN) and fusion (F) glycoproteins (9, 12, 13). Both surface proteins are necessary for viral entry into host cells. The HN protein recognizes and binds the viral receptor, and the F protein mediates viral fusion with host cell membranes (12, 13). While the HN proteins of orthorubulaviruses, such as mumps virus, are known to bind to sialic acid (12, 14, 15), pararubulaviruses, including SOSV, do not bind to sialic acid (16, 17). To date, the receptor of SOSV and other pararubulaviruses remains unknown. As the 2 glycoproteins are expressed on the surface of the viral envelope, they also are vulnerable as targets for recognition by neutralizing mAbs. The neutralizing Ab response to related paramyxoviruses is predominantly targeted to HN (18–22), but the most commonly targeted antigen recognized following natural SOSV infection and the mechanism by which SOSV-neutralizing Abs function are unknown. It is possible that structurally conserved antigenic sites exist across diverse species of rubulaviruses due to structural similarities between their F and HN proteins and the essential role of these proteins in viral fusion (12, 13).

Here, we sought to investigate the human Ab response against SOSV surface glycoproteins and identify Ab-mediated mechanisms of neutralization and breadth of reactivity of mAbs from the only known human survivor of SOSV infection. We are not aware of any previous reports of isolation of human mAbs against a rubulavirus. Such studies may contribute to a deeper understanding of human immunity to paramyxoviruses and could have direct applications in preventing or diagnosing outbreaks of SOSV. We isolated potently neutralizing mAbs from circulating memory B cells and determined the epitope specificity, binding affinity, neutralization potency, and cross-reactivity of the mAbs between diverse rubulaviruses. The data reveal key information on the neutralizing immune response to a novel paramyxovirus and identify pan-rubulavirus epitopes recognized by some human mAbs. The mAbs generated in this study also could be used as candidate prophylactic or therapeutic Abs, diagnostic reagents, or research tools for future studies on SOSV and related viruses.

## Results

### Cell surface expression of recombinant HN and F SOSV protein antigens.

**Figure 1:**
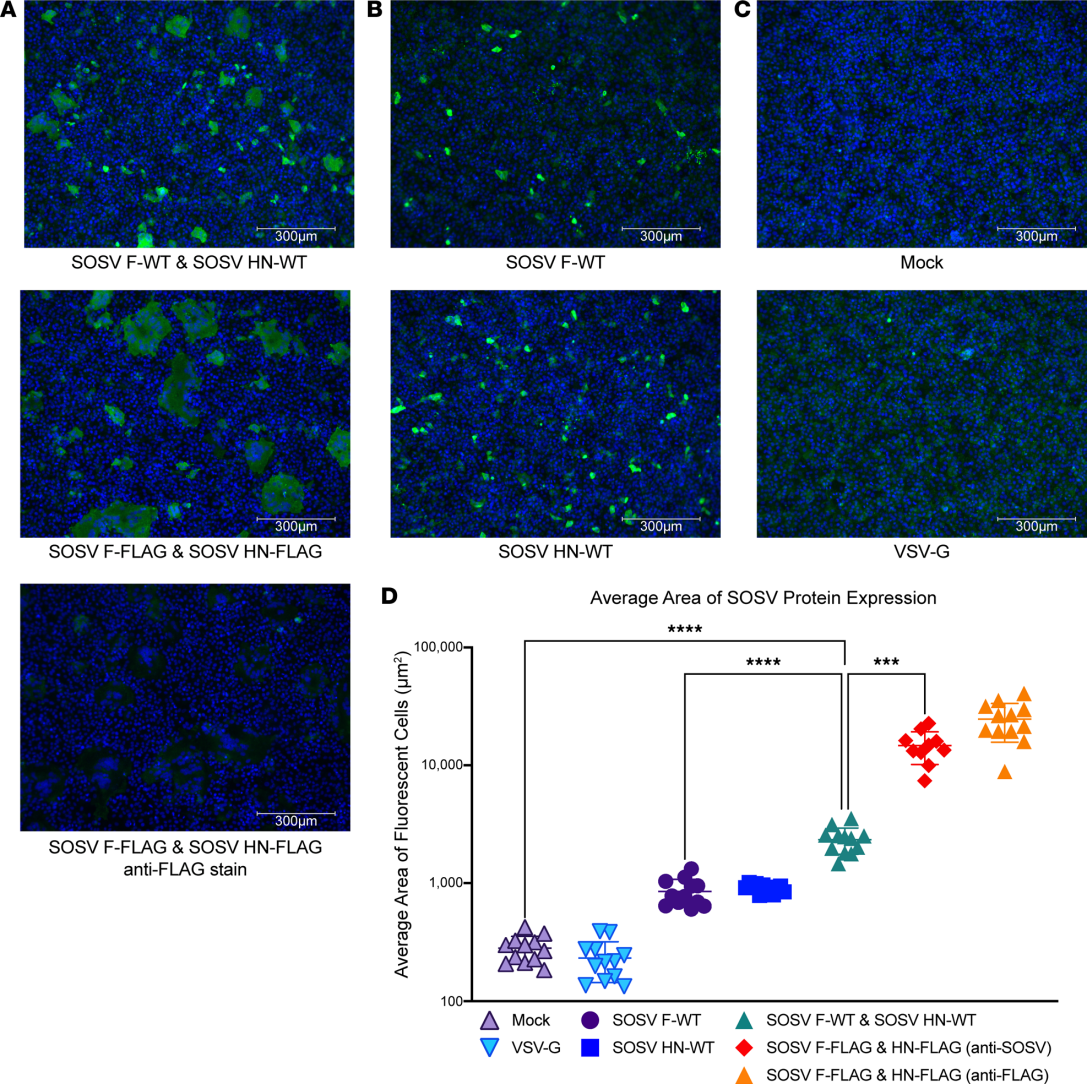
Cotransfection of cDNAs encoding SOSV F and HN proteins causes robust syncytia formation in cell culture monolayers. Representative field of view (10× objective) of transfected Vero cell culture monolayers. Nuclei were stained with DAPI (blue) and SOSV proteins were stained with a polyclonal mix of 6 anti-SOSV mAbs (3 anti-HN and 3 anti-F) or mouse anti-FLAG Ab with goat anti-human IgG with Alexa Fluor 488 dye or goat anti-mouse IgG with Alexa Fluor 488 dye Abs as secondary Abs. A total of 10–12 fields of view were imaged for each of the transfection conditions and the average area of fluorescent cells was determined for each field. (**A**) Syncytia-producing transfections: cotransfection of SOSV F-WT + SOSV HN-WT, cotransfection of SOSV F-FLAG + SOSV HN-FLAG, or cotransfection of SOSV F-FLAG + SOSV HN-Flag constructs stained with anti-FLAG Abs. (**B**) Nonsyncytia-producing transfections: cDNA-encoding SOSV F-WT or SOSV HN-WT were transfected individually. (**C**) Controls: mock transfection or VSV G-WT transfection. Scale bar: 300 μm. (**D**) Average area of fluorescently stained clusters (cells or syncytia). A 1-way ANOVA with Tukey’s multiple comparison with a *P* value threshold of < 0.05; ****P < 0.0001 and ***P < 0.001.

WT protein-encoding sequences for the 2 major surface proteins of SOSV (F and HN) were synthesized as cDNA and cloned into a mammalian expression vector (pTwist-CMV) by Twist. These constructs were transfected singly or in combination into Vero cells, and by 48 hours, visible syncytia were observed in the wells cotransfected with F and HN but not in untransfected cells or wells containing cells transfected with only HN-encoding or only F-encoding plasmids (Figure 1). Syncytium formation mediated by HN + F expression suggested both proteins were expressed on the cell surface and in a functional state.

### Expression of recombinant HN SOSV antigens for production of soluble proteins.

**Figure 2:**
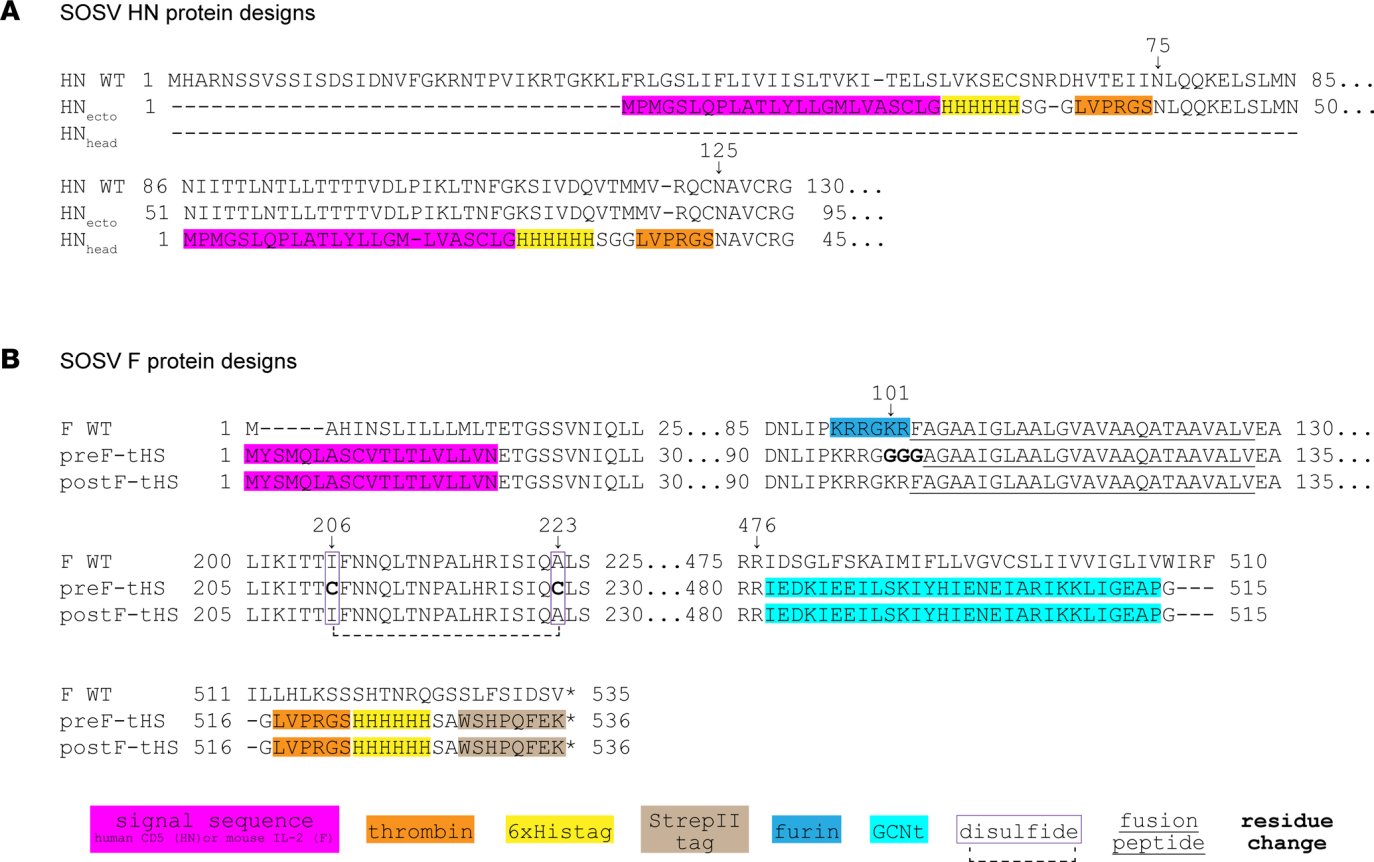
SOSV HN and F soluble protein designs. Soluble versions of the SOSV HN and F proteins were generated by removing the cytoplasmic tail and transmembrane domains and adding a human CD5 or mouse IL-2 signal peptide. To aid in purification, a 6-His tag or StrepII tags were added to the carboxy (F) or amino (HN) terminal ends. (**A**) HN_ecto_ design includes a longer portion of the stalk region starting at residue 75, while the HN_head_ construct is composed of nearly only the globular head domain of the HN protein. (**B**) Additional modifications were necessary to include in the SOSV F prefusion (preF-tHS) construct, which included removal of the furin cleavage site and creation of a stabilizing disulfide bond by point mutations to cysteines at 206 and 223. The postfusion construct of SOSV F (postF-tHS) more closely resembles the WT sequence but with replacement of the cytoplasmic and transmembrane domains with a GCNt trimerization domain (also in preF-tHS).

We developed expression systems for soluble SOSV HN proteins by truncating the WT HN sequence to encode aa residues 75 to 582 comprising the HN protein ectodomain (designated HN_ecto_; GenBank accession OQ384117) or residues 125 to 582 comprising the HN protein head domain (designated HN_head_; GenBank accession OQ384118) (Figure 2A), adding a CD5-signal peptide sequence and thrombin-cleavable 6-His tag to the amino terminus of the proteins. These constructs were sequence-optimized for human cell expression, synthesized as cDNA, and subcloned into the pTwist-CMV mammalian expression vector (Twist Biosciences). Soluble HN proteins were expressed in Expi293F cells for 5–7 days and purified from cell supernatants using an ÄKTA pure system (Cytiva), and the eluate was concentrated and buffer exchanged into Dulbecco’s phosphate-buffered saline (DPBS). As expected, the final purified proteins were about 70 kDa in apparent molecular weight.

### Expression of recombinant F SOSV antigens for production of soluble proteins.

We designed soluble forms of the SOSV F protein in both prefusion-stabilized and postfusion conformations (Figure 2B) and cloned the corresponding synthesized cDNAs into the pVRC4800 vector (GenBank accessions OQ384119 and OQ384120, respectively). Both the pre- and postfusion F protein constructs contain a mouse IL-2 signal sequence, the SOSV F protein sequence from residues 15 to 476, and an engineered, trimeric coiled-coil domain of the yeast transcriptional activator GCN4 leucine zipper (GCNt; ref. 23), replacing the transmembrane and cytoplasmic domains. Additionally, the prefusion design disrupted the polybasic furin cleavage site by replacing 3 residues (from 101–103) with a soluble glycine linker and adding a stabilizing disulfide bond between residues 206 and 223. Soluble protein was generated by expressing the constructs in Expi293F cells for 5 days before purification on the ÄKTA pure system (Cytiva) or AZURA P 6.1L (Knauer).

### Isolation of SOSV-reactive mAbs.

Leukapheresis samples from the only known human case of SOSV infection were obtained 5 years after infection. PBMCs were isolated and transformed with EBV to form lymphoblastoid cell lines, which were expanded, fused with a nonsecreting myeloma cell partner to make stable hybridoma cell lines, and single-cell–sorted using flow cytometry to isolate biologically cloned hybridoma cell lines secreting mAbs. Throughout this process, cell supernatants were screened for binding to F or HN cell surface–expressed SOSV glycoproteins to identify the cell lines containing SOSV-specific IgG-secreting B cells. In total, 24 SOSV-reactive mAbs (GenBank accession numbers OQ384121 through OQ384168) were isolated, with 18 binding to F protein and 6 binding to HN protein (Table 1 and Table 2). The Abs are predominantly of the IgG1 subclass, with 2 mAbs (SOSV-2 and -32) being IgG3. Most (19 of the 24) mAbs use a κ light chain, while 5 clones (SOSV-13, -21, -64, -83, and -85) use a λ_1_ or λ_2_ light chain. Recombinant versions of each member of the mAb panel were created by synthesizing a cDNA encoding the Ab variable gene regions and cloning by Gibson assembly into a human IgG1 expression vector (with κ or λ light chain as appropriate). The recombinant mAbs (rmAbs) each exhibited the ability to bind the appropriate cell surface–displayed viral antigen, as expected, and were used in all assays in this work.

### Domain specificity of HN-reactive mAbs.

**Figure 3:**
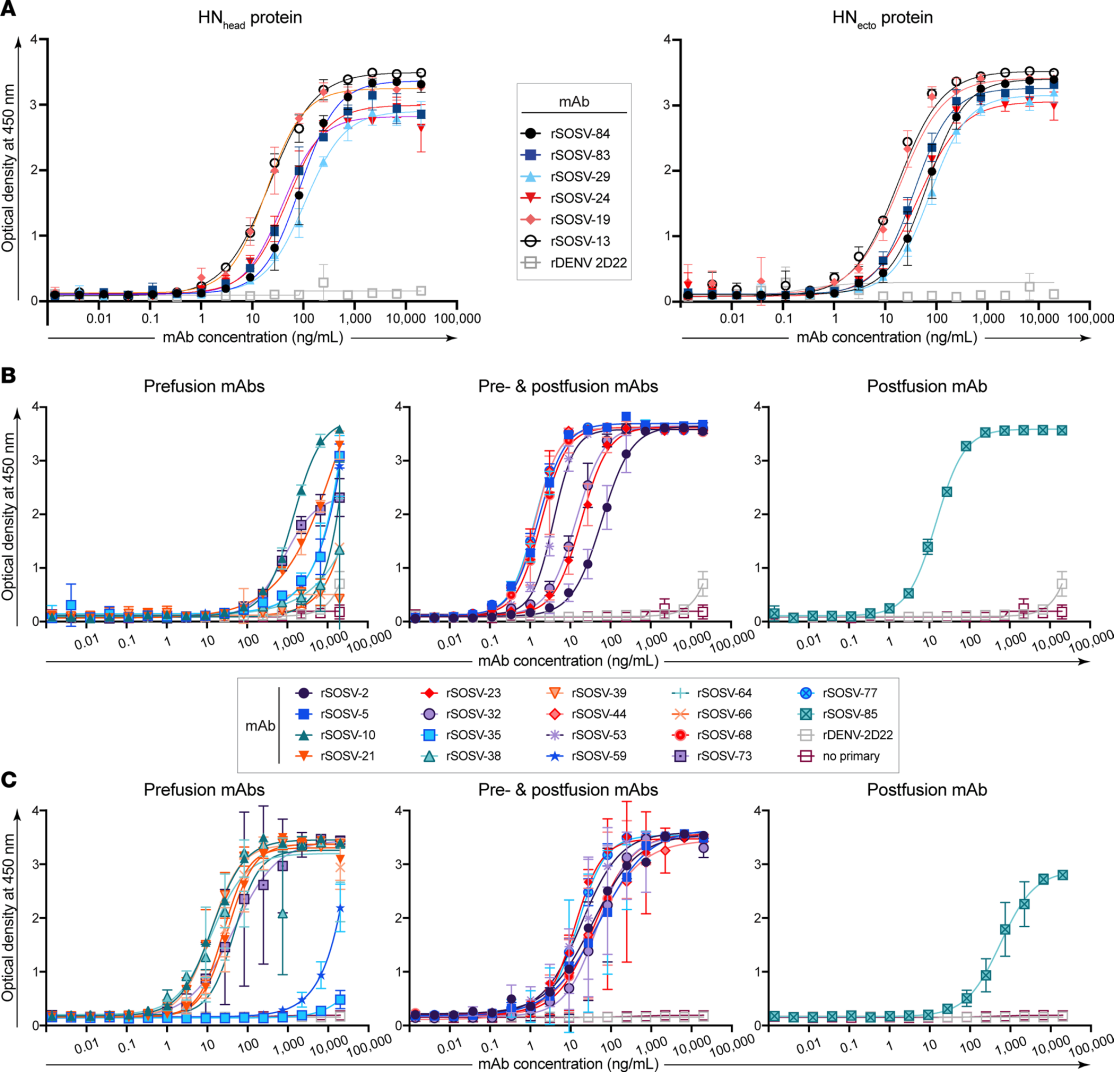
Representative curves in ELISA for binding of rSOSV mAbs to soluble glycoprotein antigens. Shown here is a single representative set of data of the 3 biological replicates that were performed. Curves show the average of 3 technical replicates plotted with SD. The anti-F rmAbs are divided into 3 subsets: prefusion, pre- and postfusion, or postfusion, although all the rmAbs were tested simultaneously. (**A**) Anti-HN rSOSV mAbs against HN_ecto_ and HN_head_ proteins. (**B**) Anti-F rSOSV mAbs against postfusion SOSV F. (**C**) Anti-F rSOSV mAbs against SOSV prefusion F construct.

**Figure 4:**
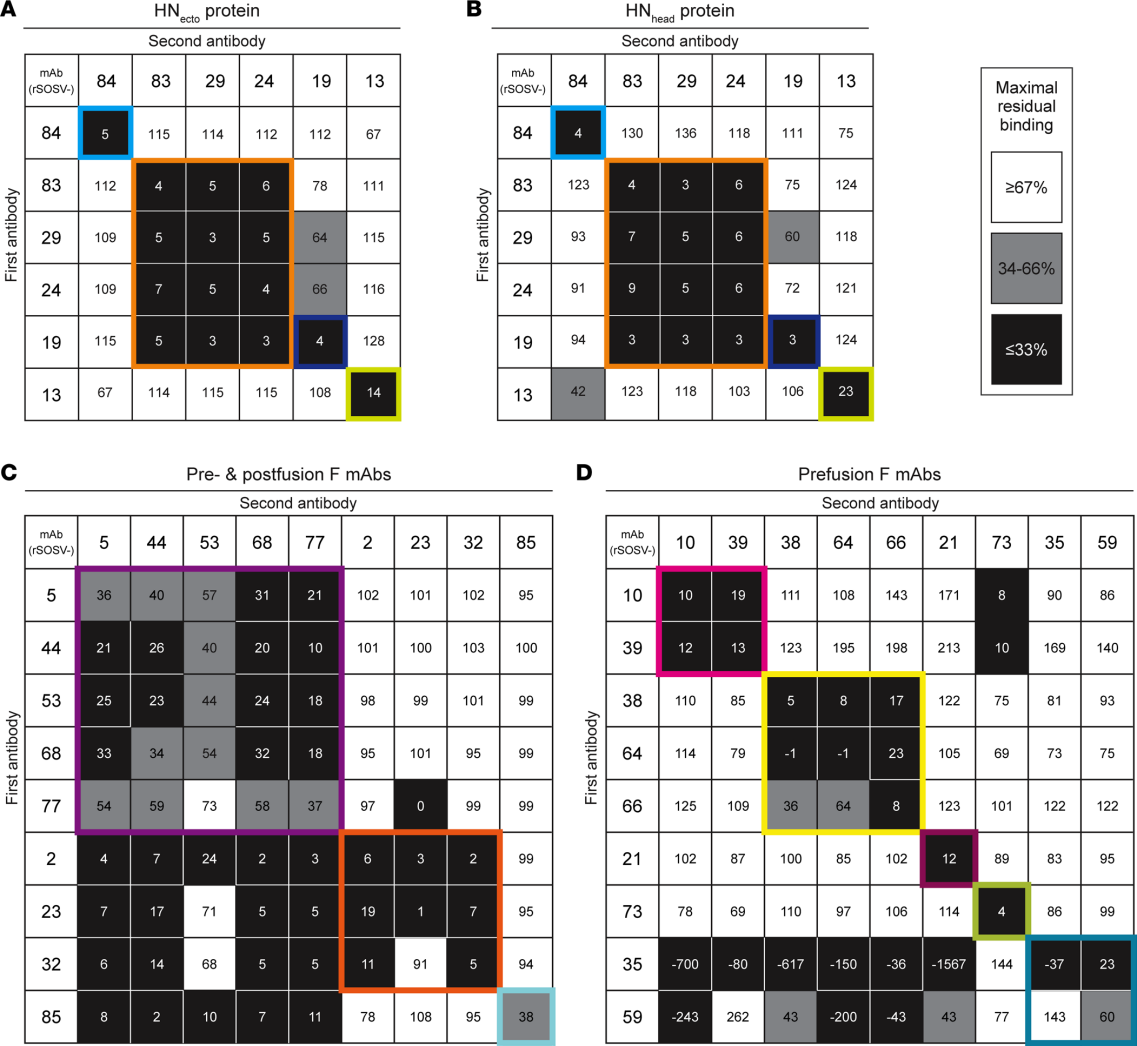
Competition-binding assay for anti-HN mAbs. Unlabeled primary (first) mAb was bound to antigen-coated plates in saturating conditions (10 μg/mL) with secondary Abs added at a final concentration of 100 ng/mL for HN-mAbs and 500 ng/mL for F-mAbs according to the grid layout shown. Data were converted to percent binding relative to the maximal uncompeted binding of the second Ab (lacking a primary mAb). Assays were repeated in triplicate with quadruplicate technical replicates. A representative assay for each antigen/mAb set tested is shown. (**A**) Binding data of mAbs against HN_ecto_ as antigen. (**B**) Binding data of mAbs against HN_head_ as antigen. (**C**) Binding data for pre- and postfusion anti-F mAbs (rSOSV-2, 5, 23, 32, 44, 53, 68, and 77) and the postfusion mAb (rSOSV-85) against prefusion F protein. (**D**) Binding data for prefusion F-specific mAbs (rSOSV-10, 21, 35, 38, 39, 59, 64, 66, and 73) against prefusion F antigen.

The specificity of the HN-reactive mAbs was refined further by testing for binding of the rmAbs to the recombinant HN_head_ protein or the tetrameric/dimeric HN_ecto_ protein in ELISA. All 6 of the HN-reactive mAbs (SOSV-13, -19, -24, -29, -83, and -84) bound to both HN antigens (Figure 3), indicating that the binding sites for all mAbs were likely located in the globular head domain of the HN protein. The EC_50_ values for binding of the rmAbs to soluble HN proteins ranged from 22 to 92 ng/mL (Table 2). For comparative purposes, 2 of the mAbs (SOSV-84 and SOSV-24) were purified as IgG from hybridoma cell line supernatants and tested in ELISA; they had similar EC_50_ values for binding compared with their respective recombinant versions (not shown). The HN-reactive rmAbs were also tested in a competition-binding ELISA (Figure 4, B and C). The data revealed that there are 4 HN competition-binding groups represented in the panel, with members as follows: group 1 (SOSV-84); group 2 (SOSV-83, SOSV-29, and SOSV-24); group 3 (SOSV-19); and group 4: (SOSV-13). Four competition-binding groups were identified consistently whether testing for competition binding to HN_head_ or HN_ecto_ proteins (Figure 4, A and B).

### Conformational specificity of anti-F mAbs.

Since paramyxovirus fusion proteins are well known to only be metastable in the prefusion state (12, 13, 24), we used soluble post- and prefusion–stabilized proteins to test for conformational specificity of the panel of anti-F mAbs. Only 1 mAb (rSOSV-85) was specific to the postfusion conformation while 7 mAbs (SOSV-10, -21, -38, -39, -64, -66, and -73) were specific to the prefusion conformation (Figure 3, B and C). Eight mAbs (SOSV-2, -5, -23, -32, -44, -53, -68, and -77) recognized both the post- and prefusion proteins (Figure 3, B and C). Two mAbs, rSOSV-35 and rSOSV-59, did not bind detectably to either pre- or postfusion soluble F protein in ELISA (Figure 3), although they did bind to cell surface–displayed WT protein and were included as the prefusion-specific element. The relative EC_50_ values for binding of the rmAbs are summarized in Table 1 and range from 1.5 ng/mL to over 4,000 ng/mL against postfusion F and 15.7 ng/mL to over 1,000 ng/mL for prefusion F. As with the anti-HN mAbs, we further categorized the anti-F mAbs by competition-binding ELISA (Figure 4, C and D) on their respective SOSV F conformational specificity. The data revealed that there are approximately 8 distinct competition-binding groups: group 1 (SOSV-73); group 2 (SOSV-66, -64, and -38); group 3 (SOSV-39 and SOSV-10); group 4 (SOSV-21); group 5 (SOSV-59 and SOSV-35); group 6 (SOSV-77, -68, -53, -44, and -5); group 7 (SOSV-32, -23, and -2); and group 8 (SOSV-85).

### Neutralizing activity of human mAbs against authentic SOSV.

**Figure 5:**
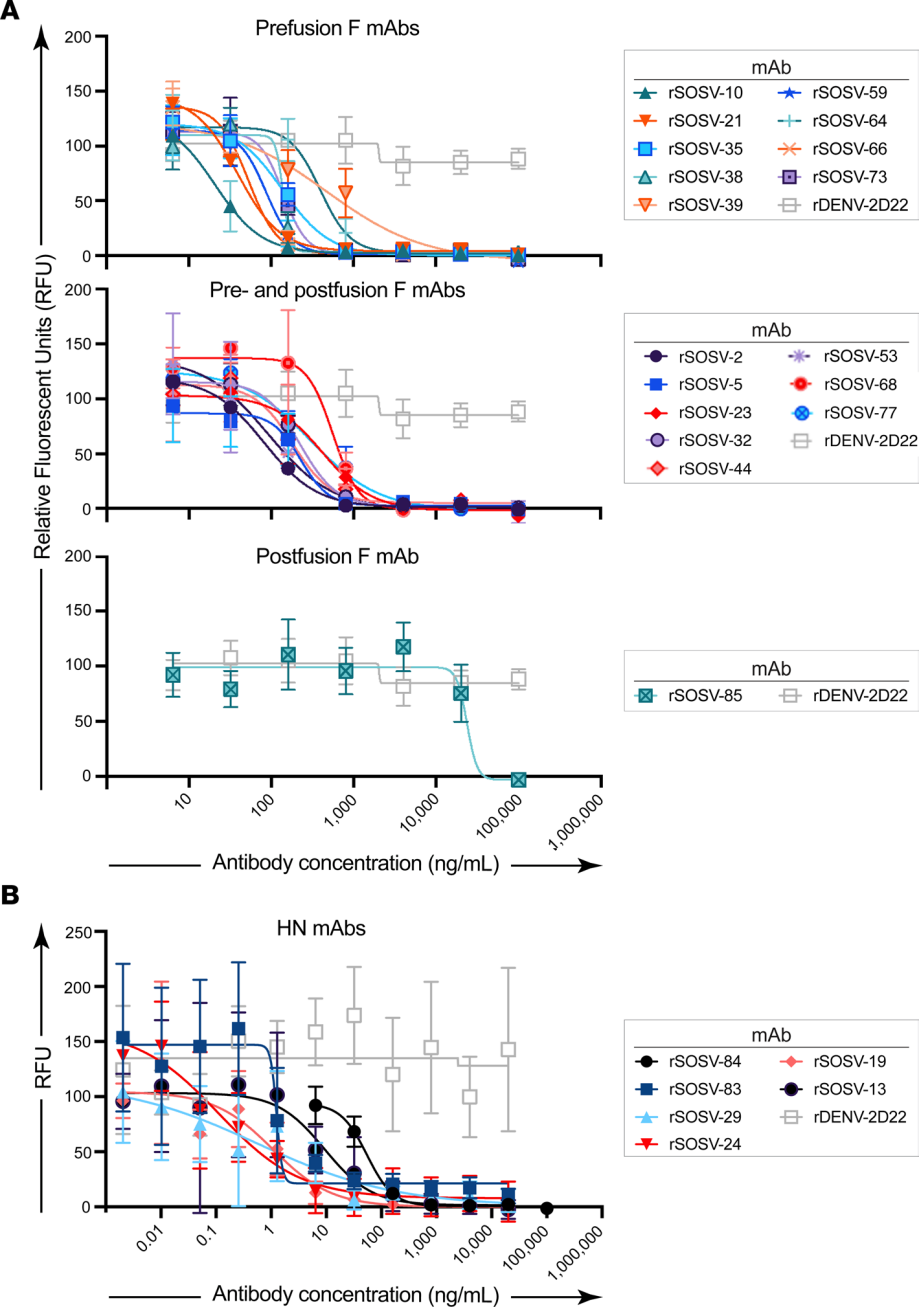
Neutralization assay of SOSV mAbs against live virus. SOSV mAbs were tested for inhibition of authentic rSOSV-ZsG in quadruplicate on Vero-E6 cell culture monolayers. (**A**) Neutralization data for anti-F mAbs. Data are grouped according to the pattern of antigen reactivity: prefusion F, pre- and postfusion F, or postfusion F protein. (**B**) Neutralization data for the HN-specific mAbs.

We next tested each of the 24 human mAbs for neutralization against recombinant SOSV expressing ZsGreen (25) by incubating dilutions of mAb with SOSV and adding to Vero cell culture monolayers. Quantification of the ZsGreen reporter was used to quantitate SOSV infection in the monolayer (Figure 5) and calculate the IC_50_ value for each mAb (Table 3). Each of the mAbs inhibited virus infection, except for the postfusion-specific mAb. As a class, the HN-specific mAbs neutralized more potently than the anti-F mAbs. Of the anti-F panel, the prefusion-specific mAbs neutralized more potently. Even though this individual experienced a primary infection with a novel paramyxovirus, the infection induced some Abs with ultrapotent (<1 ng/mL) neutralizing activity, which are some of the most potently neutralizing human Abs to a virus ever reported.

## Discussion

These studies of human Ab responses to SOSV are of interest because they elucidate the molecular basis for response to an emerging virus in the *Pararubulavirus* genus, which is understudied. The global virome, especially the virome of bats in the wild, contains many viruses that have epidemic potential (26), but paramyxoviruses are perhaps the most common threats (27). Little is known of the determinants of immunity for most of these viruses. There is a licensed vaccine for mumps virus, which is a prototype virus in the *Orthorubulavirus* genus, but the mechanisms by which this vaccine protects and the correlates of protection against mumps virus infection and disease are not defined. Here, we surveyed the antigenic landscape on the 2 major surface proteins of SOSV to understand the principal determinants of immunity for a virus in this genus. We isolated naturally occurring human mAbs that recognized HN or F proteins since other paramyxoviruses’ attachment and fusion proteins usually contain sites of vulnerability for neutralization. Before this work, it was not clear if SOSV HN and F proteins induce neutralizing Abs, and if so, which is the more immunodominant protein and what regions on these proteins induce neutralizing Abs.

Of the 6 anti-HN mAbs isolated, each recognizes sites on the globular head domain of HN. Even though all the HN-reactive mAbs target the head domain, the competition-binding patterns reveal that there are at least 4 distinct major antigenic sites recognized by these mAbs. Most of the Abs we isolated from this individual neutralized authentic SOSV, and some of them did so with very potent activity (exhibiting very low IC_50_ values in in vitro neutralization assays). Since the HN protein is the principal attachment factor for paramyxoviruses, anti-HN SOSV mAbs might be expected to possess more potent neutralizing activity than F protein–specific mAbs, and indeed we found this to be the case. The high neutralizing potency of the group 2 anti-HN mAbs suggests that these Abs may block HN protein binding to cell surface receptors. It is possible that the prolonged and marked viremia of the wildlife researcher induced ultrapotent antibodies that may have contributed to the survival of this individual but might not be elicited as commonly in humans during mild infection. An unanswered question is the seroprevalence of SOSV in people living in South Sudan or Uganda where Egyptian rousette bats are present, as well as whether SOSV infection in people living in areas endemic with pararubulaviruses would have the same susceptibility to severe disease as this wildlife researcher of US origin. The receptor for SOSV is not known, but future studies of these mAbs may help in efforts to discover the receptor for SOSV and possibly related pararubulaviruses. Blocking attachment is often one of the most efficient and complete mechanisms of Ab-mediated neutralization. However, this is not always the case since Abs to pneumovirus fusion proteins are often more potent as a class than Abs to the attachment protein for those viruses (for example, Abs to respiratory syncytial virus or human metapneumovirus F and G surface glycoproteins) (28). Additionally, blocking the HN and F proteins from interacting should inhibit successful fusion even if HN successfully attaches to the receptor, as HN is thought to trigger the conformational change in F to facilitate virus-cell fusion (12, 24, 29, 30). Particularly, the stalk domain of HN is thought to be the region that interacts with the F protein (24). Since all the anti-HN mAbs isolated in this assay bind to the head domain, it is possibly beneficial to conduct additional discovery screening to isolate stalk-specific mAbs that may neutralize through inhibiting HN-F interactions. Paramyxoviruses using proteinaceous receptors tend to have more conservation in their stalk domains than paramyxoviruses using sialic acid (31); therefore, mAbs targeting the SOSV HN-stalk region may also be more broadly reactive than mAbs binding to the head domain.

The F protein of paramyxoviruses can exist in 2 very distinct conformations, prefusion and postfusion. Most postfusion-specific F Abs are expected to be non-neutralizing or poorly neutralizing since virions particles possess the prefusion form of F, and the postfusion state occurs following viral fusion with host cell membranes. The prefusion conformation of the F protein, however, is a potential neutralizing target since Abs that block the triggering of the prefusion F protein may prevent virus-cell fusion. We tested our F-specific mAbs for their ability to recognize pre- or postfusion F proteins and found that we could classify our panel into 3 broad groups: mAbs that recognized 1) prefusion, 2) postfusion, or 3) both conformations. As expected, the prefusion-specific mAbs as a group tended to have higher neutralization potency (i.e., lower IC_50_ values) compared with the postfusion-specific mAbs and many of the pre- and postfusion-specific mAbs. Of the panel of 24 mAbs, only the postfusion-specific mAb failed to neutralize virus. The SOSV survivor’s potent Ab response at 5 years after infection (presumably without reexposure to virus while living in the United States) to both F and HN glycoproteins is remarkable and suggests the possibility for inducing durable vaccine-induced durable functional immunity in humans should the need ever arise.

For our cell surface display assay, we theorize that the WT F protein is predominantly in the prefusion state due to the absence of coexpression of the HN protein (11, 23–25). This idea is supported by the lack of syncytia formation (Figure 1B) and that only 1 of the 18 anti-F mAbs was postfusion specific. An advantage of the cell surface display system used here is that it displays the entire, WT glycoprotein, which led to the discovery of the anti-F mAbs rSOSV-35 and rSOSV-59. Neither of these mAbs bind well in ELISA assays to either conformation of the F protein (Figure 3, B and C); however, they are quite potent neutralizers with IC_50_ values of 87–137 ng/mL (Table 3) and bind in cell surface display. Since it is likely that cell surface–displayed F is in the prefusion state, we categorized rSOSV-35 and rSOSV-59 as prefusion specific. Both mAbs also fall into the same competition-binding group as tested by ELISA (Figure 4, C and D) and verified through cell surface display (data not shown). Due to the poor binding in ELISA, it is likely that the epitope(s) for these 2 mAbs is absent or obstructed in the soluble designs of the F protein. Had ELISA assays been used from the start of Ab discovery, then, it is quite likely that Abs such as rSOSV-35 and rSOSV-59 would not have been isolated. Thus, using ELISA-based screening to identify novel paramyxoviruses mAbs may miss discovery of some strongly neutralizing Abs. Future Ab discovery campaigns for other novel paramyxoviruses like SOSV may then benefit from the cell surface display assay described here. This screening method allows for the entirety of the glycoprotein to be expressed and appears to better keep the metastable F protein in the preferred neutralization state (prefusion) than the soluble prefusion design described here (Figure 2B), so long as HN is not coexpressed.

This panel of mAbs may be useful in several applications. An ultrapotent HN mAb, such as rSOSV-24, is potentially a therapeutic candidate given its extraordinarily low IC_50_ value for neutralization of 0.4 ng/mL. As there are currently no available SOSV-specific reagents, the mAbs discovered in this work also can serve as reagents for the continued study of SOSV pathogenesis and immunity. rSOSV-85, as a postfusion-specific mAb, can be used in various applications, such as to study the fusion-triggering process during the SOSV life cycle, help test the stability of potential prefusion F protein vaccine candidates, or aid in prefusion F protein purification processes by sequestering postfusion F protein during chromatographic purification protocols. Potently neutralizing prefusion-specific F mAbs like rSOSV-10 can serve as positive controls in neutralization assays for testing antiviral compounds or vaccines. Finally, knowledge of the competition-binding groups of the HN and F proteins may help in further understanding protein domains governing the paramyxovirus fusion process or in receptor discovery studies for SOSV. As SOSV and other pararubulaviruses lack the ability to bind to sialic acid but can infect human cells (16, 17), discovering the receptor for this genus of paramyxoviruses could greatly advance efforts for epidemic preparedness against this group of viruses. Also, since all the pararubulaviruses tested so far (Teviot, Tioman, and Menangle viruses) are able to enter bat, human, and pig cells (5, 7, 16, 32, 33) it is quite possible that the SOSV receptor may also be conserved between bats humans, and pigs, indicating potential threat to or from domestic livestock as well.

In summary, the human mAbs isolated in this study are the first SOSV-specific mAbs generated to our knowledge and can be used for further studies of SOSV and related viruses and as candidate therapeutic Abs for clinical development. Additionally, the methods and approaches used in this study may be beneficial for the isolation of Abs against other novel paramyxoviruses, particularly those in the *Rubulavirinae* subfamily.

## Methods

### Immune cells.

In 2012, a 25-year-old otherwise-healthy individual was infected with SOSV during occupational exposure while handling wild bats as part of wildlife surveillance activities in South Sudan and Uganda. Clinical and virologic features of infection for this only known human survivor of SOSV infection have been described previously (7). RNA extraction from the individual’s whole blood followed by reverse transcription PCR (RT-PCR) amplification and DNA sequence analysis revealed the presence of RNA genome for a novel paramyxovirus that was named *Sosuga pararubulavirus*, or Sosuga virus (SOSV), after the countries visited by the wildlife researcher (South Sudan and Uganda) (7). A leukapheresis pack was obtained from the individual after written informed consent in 2017, approximately 5 years after the infection. PBMCs were isolated from the leukapheresis product, cryopreserved at a density of 10 or 25 million cells/mL, and stored in the vapor phase of liquid nitrogen until use.

### EBV transformation of cell lines from human blood.

Vials of cryopreserved PBMCs were thawed at 37°C and washed in ClonaCell-HY Medium A (StemCell Technologies, catalog 03801). EBV was obtained by collecting the supernatant of the marmoset lymphoblastoid cell line (LCL) B95-8 (34), which was formerly available from the American Type Culture Collection (ATCC) as ATCC CRL-1612. B cells were transformed with EBV by combining washed PBMCs with prepared stocks of filtered B95-8 cell supernatant, using 4.5 mL to transform 8 million to 10 million PBMCs in B cell growth medium, made up of Medium A containing CpG (Invitrogen, oligo ZOEZOEZZZZZOEEZOEZZZT) at the 10 μmol scale (desalted), cyclosporin A (Sigma-Aldrich, catalog C1832), and Chk2 inhibitor II (Sigma-Aldrich, catalog, C3742). Cells were plated at 50 μL/well in a 384-well plate for each suspension of 8 million to 10 million PBMCs. Cells were incubated at 37°C in 7% CO_2_ for 6–12 days until LCLs were clearly visible and forming colonies. The plates of transformed B cells were expanded to four 96-well plates in B cell expansion medium (Medium A, CpG, Chk2i II, and 10 million irradiated human PBMCs per plate from an unrelated healthy donor; Nashville Red Cross). The plates were incubated at 37°C in 7% CO_2_ for 4–7 days before screening Abs in LCL supernatants for binding to SOSV antigen expressed in cells using a high-throughput flow cytometry assay.

### Production of human hybridoma cell lines from transformed B cells.

Once positive SOSV-reactive wells of LCLs were identified, the B cells from these wells were transferred to microcentrifuge tubes and washed 3 times with BTX medium, made up of 300 mM sorbitol (Thermo Fisher Scientific, catalog BP439), 0.1 mM calcium acetate (Thermo Fisher Scientific, catalog AC21105-2500), 0.5 mM magnesium acetate (Thermo Fisher Scientific, catalog AC42387-0050), and 1.0 mg/mL BSA (Sigma-Aldrich, catalog A3294). The B cell pellets were resuspended in BTX medium, combined with the HMMA2.5 human-mouse myeloma fusion partner cell line, and electroporated in a 0.2 μm cuvette (BTX, catalog 45-0125). After fusion, cells were left in cuvettes in 7% CO_2_ at 37°C for at least 30 minutes before being transferred to hypoxanthine-aminopterin-thymidine (HAT) selection medium, made up of 20% ClonaCell-HY Medium E (StemCell Technologies, catalog 03805), 80% Medium A, HAT media supplement (final concentrations 100 μM hypoxanthine, 0.4 μM aminopterin, and 16 μM thymidine; Sigma-Aldrich, catalog H0262-10VL), and 150 μL of 1 mg/mL ouabain octahydrate (final concentration 0.33 μg/mL; Sigma-Aldrich, catalog O3125). Fused cells were plated by limiting dilution in 384-well plates with 50 μL/well volumes. The plates were incubated for 2–3 weeks, feeding with 25 μL/well of Medium E after 1 week, before screening for binding to recombinantly expressed viral antigens by high-throughput flow cytometry to identify wells with hybridomas secreting SOSV-reactive Abs. SOSV-reactive hybridomas were expanded to 48-well plates with 500 μL/well Medium E and screened again.

### Isolation of human mAbs secreted from hybridoma cell lines.

The hybridoma cell lines secreting SOSV-reactive Abs were cloned using single-cell sorting on a BD FACSAria III cytometer or SH800 Cell Sorter (Sony Biotechnology) into 384-well plates containing Medium E and incubated in 7% CO_2_ at 37°C for 1–2 weeks for the cells to expand in number. Supernatants from 384-well plates (1 plate for each hybridoma line) were screened by high-throughput flow cytometry using cell surface–expressed SOSV antigens to identify hybridoma cell clones secreting SOSV-specific Abs. The cloned hybridoma cell lines with antigen-reactive supernatants were scaled up gradually in 48-well, 12-well, T-25, and T-75 plates or flasks with screening for Ab binding to cell surface displayed antigens by high-throughput flow cytometry at each expansion step. The cells from T-75 flasks were used to make frozen stocks of the mAb-secreting cloned hybridoma cell lines by freezing cells in freezing medium (50% cell culture medium, 40% fresh Medium E, and 10% dimethyl sulfoxide).

### Sequence analysis of Ab variable genes.

Cell pellets of the cloned hybridoma cell line cultures were processed for RNA extraction and amplification of Ab variable genes by 5′ RACE or 3′ RACE procedures, and DNA sequence analysis of cDNA using a Sequel instrument (Pacific Biosciences) as previously described (33).

### Purification of mAb proteins.

MAb IgG proteins in supernatants of cloned hybridoma cell lines were prepared by washing cells from T-75 flasks in serum-free Hybridoma-SFM Medium (Thermo Fisher Scientific, catalog 12045076) and seeding 3–6 wells of a 6-well G-Rex plate (Wilson Wolf, catalog 80240M) with the mAb-secreting lines in Hybridoma-SFM medium. The G-Rex plates were incubated in 7% CO_2_ at 37°C, with the supernatant typically being harvested and cells split every 1–2 weeks. The G-Rex wells were reseeded up to a maximum of 3 times. MAb supernatants were collected and clarified through a 0.2 μm filter, and then mAbs were isolated by fast protein liquid chromatography (FPLC) on an ÄKTA pure system (Cytiva) using HiTrap Protein G High Performance (Cytiva, catalog 17-0404-01) or HiTrap MabSelect SuRe (Cytiva, catalog 11-0034-95) columns.

### High-throughput flow cytometric detection of binding to cell-associated viral antigens.

Expi293F cells (Thermo Fisher Scientific, catalog A14527) were transfected with DNA plasmids encoding either WT full-length SOSV F (YP_009094032.1) or HN (YP_009094033.1) constructs. Cells were seeded in flat-bottomed, Erlenmeyer flasks at 2.5 × 10^6^ live cells/mL, using the volume of the culture size to set the scale of the transfection mix. The transfection mix was prepared by combining cold Opti-MEM I Reduced-Serum Medium (0.1 mL/mL of cells; Thermo Fisher Scientific), DNA (1 μg/mL of cells), and 2.7 μL/mL of cells Expifectamine 293 reagent (Thermo Fisher Scientific, catalog A14524), mixing 3–5 times by pipetting, and incubating at room temperature for 20–30 minutes. Flasks of cells first were swirled before adding the transfection mix to ensure even spreading of the transfection mix. Cells were incubated at 37°C in 7% CO_2_ with shaking at 0.22*g* for 24–48 hours. The day after transfection, ExpiFectamine 293 Transfection Enhancer 1 and Enhancer 2 were added at 0.5% or 5% scale of transfection, respectively. Transfected cells were plated at 50,000 to 70,000 live cells/well in 96-well V-bottom plates, washed with flow cytometry buffer (DPBS without calcium and magnesium, 2% low-IgG FBS, and 2 mM EDTA), and stained with Abs in the supernatants of transformed B cells or hybridoma cells, or purified mAb, at 30–50 μL/well at 4°C for 30 minutes as the primary stain. The primary stain was washed off with flow cytometry buffer, and the cells were stained with 50 μL/well of a 1:1,000 dilution of goat anti-human IgG-PE (Southern Biotech, catalog 2040-09) secondary Abs for 30 minutes at 4°C. The secondary Abs were removed by washing with DPBS, and the cells were fixed with 50–100 μL of 4% paraformaldehyde (PFA) in DPBS for 10 minutes at room temperature. For screening for binding of Abs in hybridoma supernatants or suspensions of purified mAbs, cells were stained with LIVE/DEAD Fixable Violet Dead Cell Stain (Invitrogen, catalog L34963) for 30 minutes at 4°C prior to fixation. The fixative was washed off with flow cytometry buffer, and cells were resuspended in 25 μL of flow cytometry buffer and analyzed on an iQue Screener PLUS cytometer (Sartorius).

### SOSV F and HN protein constructs.

Coding sequences for the WT SOSV F or HN proteins were obtained from the 2012 human isolate sequences (GenBank NC_025343.1), then sequence-optimized for human expression, and cDNA was synthesized by Twist Bioscience and inserted into pTwist-CMV expression vectors for use in cell surface expression assays. Additionally, constructs of the HN and F WT coding sequences were generated with the same sequences as above but with the addition of cDNA encoding a DYKDDDDK (FLAG) tag on the cytoplasmic domain of the proteins (carboxy terminus for the F protein and amino terminus for the HN protein) and synthesized by Twist Bioscience. Plasmids containing cDNAs encoding soluble forms of the HN protein were synthesized by Twist Bioscience with a CD5 signal peptide sequence for secretion and a thrombin-cleavable 6-His tag for purification. The construct for the ectodomain of the HN protein (designated HN_ecto_) contains aa residues 75 to 582, while the HN head domain construct (designated HN_head_) contains residues 125 to 582, as previously described (17). Soluble forms of the prefusion F protein were designed that included residues 15 to 476 of the SOSV F sequence with the following modifications: aa changes I206C, A223C, and K101-F103GGG were introduced, a GCNt domain was added to the C-terminus, and a mouse IL-2 signal peptide was placed into the pVRC8400 vector at the N-terminus of the protein-coding sequence. The I206C and A223C mutations create a disulfide bond, and K101-F103GGG edits the furin cleavage site.

### Data sharing of Ab and soluble antigen sequences.

Ab heavy and light chain V-gene sequence data and those for the soluble antigen designs have been deposited in GenBank with accession numbers OQ384117 to OQ384168 to aid in the reproducibility of this work (Supplemental Table 1; supplemental material available online with this article; https://doi.org/10.1172/jci.insight.166811DS1).

### Protein purification for recombinant mAbs and soluble antigens.

Plasmids encoding the recombinant mAbs were expressed using the ExpiCHO expression system (Thermo Fisher Scientific, catalog A29130). Cultures of ExpiCHO cells (Thermo Fisher Scientific, catalog A29127) were transfected at a density of around 6 × 10^6^ live cells/mL in flat-bottomed Erlenmeyer flasks following the manufacturer’s protocol. Cells were cultured at 37°C, 7% CO_2_ with shaking at 0.22*g* and harvested 8–10 days after transfection. The recombinant mAb supernatants were collected and clarified through a 0.2 μm filter, and then mAbs were isolated by FPLC on an ÄKTA pure system using HiTrap Protein G High Performance or HiTrap MabSelect SuRe columns (Cytiva). Eluants were collected and concentrated using Amicon Ultra-15 Centrifugal Filters with Ultracel-10 or Ultracel-30 Membrane (MilliporeSigma, catalog numbers UFC901024 or UFC903024, respectively), and buffer was exchanged to DPBS with Zeba Spin Desalting Columns (7K MWCO, 10 mL; Thermo Fisher Scientific, catalog 89894). Ab stocks were diluted to a concentration of 1 mg/mL, aliquoted, and flash-frozen using dry ice and ethanol bath before being stored at –80°C until needed. When thawed, Ab stocks were maintained at 4°C. Soluble forms of the SOSV antigens with were purified by using the proteins’ 6-His tag or StrepII tags and Expi293F cells and Expifectamine 293 expression system (Thermo Fisher Scientific, catalog A14525) following the same transfection protocol as expression for cell surface display. For soluble F transfections, the cells were moved to 32°C, 7% CO_2_, shaking at 0.22*g* after the enhancer addition on day 1 after transfection. Cells were harvested at 5–7 days after transfection. Transfected cell supernatant was collected and the 6-His- or StrepII-tagged antigens purified using the ÄKTA pure system with HisTrap Excel, StrepTrapHP, or StrepTrapXT columns (Cytiva) as appropriate. Eluates were run on an SDS-PAGE gel to identify samples with the target proteins. These samples were then collected and concentrated using an Amicon Ultra-15 Centrifugal Filter with Ultracel-10, Ultracel-30, or Ultracel-100 Membranes (MilliporeSigma), and the buffer was exchanged to DPBS or Tris-saline, made up of 140 mM Tris-HCl (Corning, catalog 46-031) and 20 mM NaCl (Corning, catalog 46-032-CV) diluted in deionized, filtered water and titrated to pH 8 with Zeba Spin Desalting Columns (7K MWCO, 10 mL; Thermo Fisher Scientific, catalog 89894). Protein concentrations were typically diluted to less than 1 mg/mL, aliquoted, and stored at 4°C if being used recently or –80°C after freezing in a dry ice ethanol bath for longer-term storage.

### Microscopy of SOSV F and HN expression in cultured cells.

Vero cells (ATCC, catalog CCL-81) were seeded at 20,000 live cells/well into a clear-bottomed, black, 96-well plate (Greiner Bio-One, catalog 655090) in DMEM + 10% FBS + 1% Penicillin-Streptomycin-Glutamine (Gibco). While in suspension, cells were transfected with 10 L of Lipofectamine 3000 (Thermo Fisher Scientific, catalog L3000015) transfection mix containing plasmids encoding SOSV F-WT, SOSV HN-WT, SOSV F-WT & SOSV HN-WT, SOSV F-FLAG & SOSV HN-FLAG, VSV-G, or no DNA (mock), with 10–12 replicate wells for each condition. Transfection mixes were prepared following the manufacturer’s protocol using approximately 0.15 μL of Lipofectamine 3000 reagent and approximately 100 ng total DNA per well. The plate was incubated at 37°C in 5% CO_2_ for 45 hours, after which the medium was removed, and the cells were fixed in 100 μL of 4% PFA for 1 hour. Cells were washed several times with DPBS before blocking and permeabilizing with 150 μL/well of permeabilization buffer (5% milk and 0.1% saponin in 1 PBS with Tween 20 (PBST, 20 stock solution used to make 0.05% Tween 20 when diluted to 1; Cell Signaling Technology, catalog 9809S) or 60 minutes at room temperature. A polyclonal mix of anti-SOSV mAbs was made by mixing 3 HN mAbs and 3 F mAbs; the anti-SOSV mix was diluted to 1:250 in permeabilization buffer while monoclonal anti-FLAG M2 (Sigma-Aldrich, catalog F3165) was diluted to 1:500 in permeabilization buffer. Cells were stained with 50 μL of anti-SOSV or anti-FLAG primary (half the plate) and incubated for approximately 1 hour at room temperature, after which the primary stain was removed and the plate washed with DPBS. The cells were then stained with 50 μL of the secondary mix (goat anti-human IgG–Alexa Fluor 488; SouthernBiotech, catalog 2040-30) and goat anti-mouse Alexa Fluor 488 IgG (H + L) (Invitrogen, catalog A11001), both diluted to 1:1,000 in permeabilization buffer and incubated 1 hour at room temperature protected from light. Cells were then washed with DPBS before being stained with 50 μL/well of DAPI (Invitrogen, catalog D1306) diluted to 5 μM in DPBS for 15 minutes. Cells were then washed several times and then kept in 250 μL/well of DPBS for imaging. Imaging was done on an EVOS M5000 instrument (Invitrogen, catalog AMF5000) with a 10× objective with 4 fields of view imaged for 3 replicate wells of each transfection condition. The area of stained cells/syncytia was measured using Fiji (35), and the data were analyzed in Prism (GraphPad Software, version 9.3.1 for Mac).

### ELISA to detect Ab binding to viral proteins.

ELISAs were performed by coating 384-well plates with either soluble viral glycoprotein (HN_ecto_, HN_head_, prefusion F with 6-His and StrepII tags [preF-tHS], or postfusion F with 6-His and StrepII tags [postF-tHS] at 2 μg/mL in 20–25 μL of DPBS). Plates were coated with antigen overnight at 4°C, washed 3 times with PBST using an EL406 plate-washer dispenser instrument (BioTek), then blocked for 1–3 hours at room temperature with 50 μL/well of blocking buffer (2% Blotting Grade Blocker; Bio-Rad catalog 1706404 and 2% heat-inactivated goat serum; Gibco, catalog 16210-072) in PBST. SOSV HN mAbs were diluted in blocking buffer starting at 20 μg/mL in a 3-fold serial dilution series. After removing the blocking buffer, primary Abs were added at 20 μL/well to the plates and incubated at room temperature for 1 hour. Plates were washed 3 times with PBST prior to addition of the secondary Abs. The secondary Ab solution was prepared by diluting goat anti-human IgG HRP-conjugated Abs (SouthernBiotech, catalog 2040-05) at 1:2,000 in blocking buffer and adding 20 to 25 μL/well, and then incubated at room temperature for 1 hour. Secondary Abs were removed, and plates washed 3 times with PBST. A volume of 25 μL/well of 1-step Ultra TMB-ELISA Substrate Solution (Thermo Fisher Scientific, catalog PI34029) was added to the plates and incubated for 5–10 minutes at room temperature before being quenched with 25 μL of 1N hydrochloric acid (Thermo Fisher Scientific, catalog SA48-1). Plates were analyzed on a BioTek plate reader at 450 nm wavelength. Data were analyzed in Prism (GraphPad Software, version 9.3.1 for Mac) using a sigmoidal, 4-parameter logistic, nonlinear regression model to generate the graphs and EC_50_ values for the mAbs.

### Biotinylation of SOSV-specific Abs.

SOSV F- or HN-reactive mAbs and a similarly prepared human mAb (rDENV-2D22) specific for an unrelated virus antigen (dengue virus envelope protein) were biotinylated. Purified IgG mAb proteins were diluted to a concentration of 1 mg/mL in DPBS, and an aliquot of 200 μL volume (containing 200 ng of Ab) was used for biotinylation. A 2 mg vial of EZ-Link NHS-PEG4-Biotin, No-Weigh Format biotin (Thermo Fisher Scientific, catalog A39259) was reconstituted with 170 μL of DPBS or dimethyl sulfoxide and 1.33 μL of the biotin solution and then added to 200 ng of each of the purified Abs. Ab-biotin solutions were mixed and incubated at room temperature for 50 minutes. Excess biotin was removed using Zeba Spin Desalting Plates, 7K MWCO (Thermo Fisher Scientific, catalog 89807). The plate columns were equilibrated with DPBS following the manufacturer’s protocol. The Ab-biotin mixtures were loaded onto 2 columns for each mix, with approximately 100 μL loaded onto each column. The resulting duplicate eluates were combined.

### Competition-binding studies using ELISA.

Competition ELISAs for the anti-SOSV mAbs were performed by coating 384-well plates overnight at 4°C with 20 μL of 2 μg/mL concentration solutions of antigen in DPBS: HN_ecto_ or HN_head_ protein for anti-HN mAbs and prefusion or postfusion F protein for anti-F mAbs. Plates were washed 3 times with PBST using an EL406 plate washer (BioTek) then blocked for 1–3 hours at room temperature with 50 μL/well of blocking buffer (HN: 5% Blotting Grade Blocker, Bio-Rad, catalog 1706404 in PBST; or F: 2% Blotting Grade Blocker and 2% goat serum, Gibco, catalog 16210-072 in PBST). Blocking buffer was removed by washing plates 3 times with PBST on an EL406 plate washer. The SOSV mAbs or control mAb DENV-2D22 were diluted to a concentration of 10 μg/mL in respective blocking buffers, and 20 μL of each mAb was plated into wells of a 384-well plate to give quadruplicate readings for each mAb combination. To determine the maximal binding of each mAb in the absence of competition, 20 μL of plain blocking buffer (without a primary Ab) was placed into enough wells of a 384-well plate to give quadruplicate readings for each mAb combination. Plates were incubated at room temperature for 1 hour. The biotinylated HN mAbs were diluted to 500 ng/mL in blocking buffer while the F mAbs were diluted to 2,500 ng/mL in blocking buffer, and 5 μL of biotinylated mAb was added to the 20 μL of unlabeled mAb or blocking buffer control so that the final concentration of biotinylated Ab was 100 ng/mL for anti-HN mAbs and 500 ng/mL for anti-F mAbs. Plates were incubated at room temperature for 1 hour, then were washed 3 times with PBST using an EL406 plate washer. A volume of 25 μL of a 1:1,000 dilution of Mouse Anti-Biotin-AP (SouthernBiotech, catalog 6404-04) in blocking buffer was added to each of the wells of the HN plates and incubated for 1 hour at room temperature. For the F-coated plates, 25 μL of a 1:2,000 dilution of avidin-peroxidase (Sigma-Aldrich, catalog A7419-2ML) in blocking buffer was added to each of the wells of the plates and incubated for 1 hour at room temperature. The AP-labeled Ab or avidin-peroxidase was removed with 3 washes of PBST. For HN plates, 25 μL of phosphatase substrate (Sigma-Aldrich, catalog S0942) was diluted to 1 mg/mL in AP-substrate buffer, comprising pH 9.6, 1 M Tris Base (Tris [Hydroxymethyl] Aminomethane; Research Products International, catalog T60040) and 0.3 mM MgCl_2_ (Sigma-Aldrich, catalog M1028), and added to each well. Plates were developed in the dark at room temperature for 1 hour before being read on a BioTek plate reader at 405 nm wavelength. For the F plates, 25 μL/well of 1-step Ultra TMB-ELISA Substrate Solution (Thermo Fisher Scientific, catalog PI34029) was added to the plates and incubated for 5–10 minutes at room temperature, before being quenched with 25 μL of 1N hydrochloric acid (Thermo Fisher Scientific, catalog SA48-1) and read on a BioTek plate reader at 450 nm wavelength. Since some anti-F mAbs (rSOSV-10, 21, 35, 38, 39, 59, 64, 66, and 73) had shown poor binding to postfusion F, these mAbs were not tested for competition binding on the postfusion F protein. However, anti-F mAbs rSOSV-2, 5, 23, 32, 44, 53, 68, 77, and 85 were tested on both prefusion and postfusion F. The values obtained from quadruplicate wells were averaged, and the values from the wells with the negative control mAb rDENV-2D22 were considered the nonspecific binding signal and subtracted. The averaged absorbance data then were converted to percentages relative to the maximal (without unlabeled primary mAb) data. Competing mAbs were defined as having a residual binding level equal to or below 33% of the maximal binding level, the intermediate competition was defined as having 34%–66% of the maximal binding, and noncompeting mAbs were defined as having equal to or greater than 67% of maximal binding.

### Neutralization of SOSV by human mAbs.

The anti-SOSV mAbs were tested for neutralization activity with authentic virus under biosafety level 3 (BSL-3) conditions. To assist in viral quantification, we used a previously described recombinant SOSV-encoding ZsGreen (ZsG) protein (26). Neutralization activities of mAbs were measured using a standard protocol in Vero-E6 cell (ATCC, catalog CRL-1586) monolayer cultures. Briefly, serial 5-fold dilutions of mAbs (150 μL) made in DMEM were mixed with an equal volume of a suspension of SOSV-ZsG containing 100 median tissue culture infectious doses. After incubation at 37°C for 1 hour, 50 μL of virus-Ab mixture was inoculated onto each well containing a Vero-E6 cell monolayer culture in 96-well plates (Cell Carrier Ultra plates; PerkinElmer, catalog 6055308) that had been seeded the day before at 15,000 cells per well. The culture was incubated at 37°C for 72 hours, after which fluorescence intensities were determined using a multi-well plate reader (Synergy; BioTek). Fluorescence readings were taken from quadruplicate wells at each mAb concentration. Background fluorescence signals (obtained from wells lacking virus) were deducted from the virus and treatment readings, and data are presented as the percentage of the no-Ab and virus-only control. Prism software (GraphPad) was used to generate concentration-response plots. A 4-parameter equation was used to fit semi-log plots of the data and derive the relative IC_50_ values.

### Statistics.

A 1-way ANOVA followed by a Tukey’s multiple comparisons test was used in Prism (GraphPad Software, version 9.3.1 for Mac) to analyze the differences in cell average area of the fluorescent cells transfected with control conditions or SOSV glycoproteins (Figure 1). The *P* value threshold used was < 0.05.

### Study approval.

The studies were approved by the Vanderbilt University Medical Center Institutional Review Board. Leukapheresis samples from the individual with prior SOSV infection were obtained following informed written consent.

## Author contributions

HMP, PSR, SJ, CGA, and JEC planned the experiments. HMP, NK, EA, LH, SD, JR, JD, PSR, and SJ performed the experiments. GSJ designed the SOSV F constructs and provided reagents. CGA, RHC, and JEC supervised the research. JEC obtained funding.

## Supplementary Material

Supplemental data

## Figures and Tables

**Table 1:**
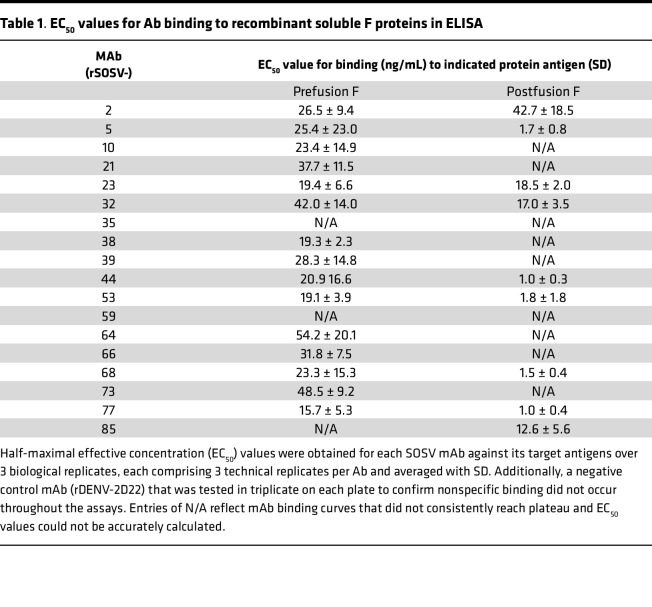
EC_50_ values for Ab binding to recombinant soluble F proteins in ELISA

**Table 2:**
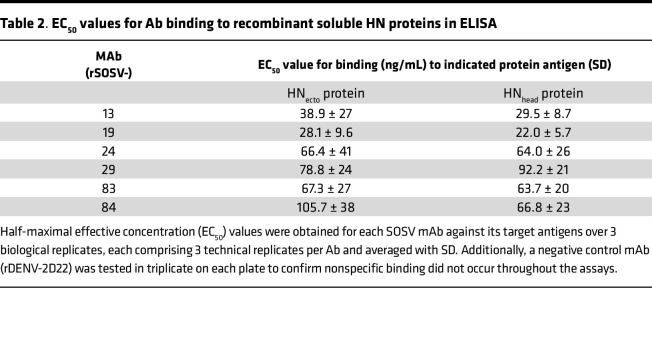
EC_50_ values for Ab binding to recombinant soluble HN proteins in ELISA

**Table 3:**
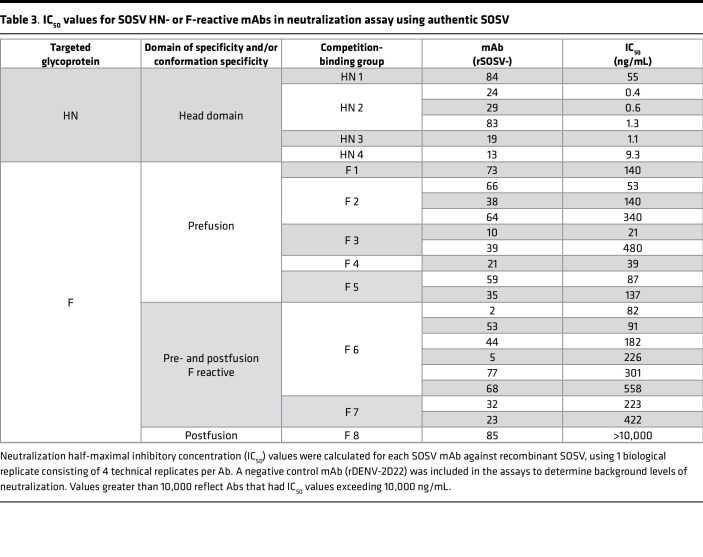
IC_50_ values for SOSV HN- or F-reactive mAbs in neutralization assay using authentic SOSV

## Version 1 02/28/2023

In-Press Preview

## Version 2 04/24/2023

Electronic publication
