# Supplementary material for: Potently neutralizing human mAbs against the zoonotic pararubulavirus Sosuga virus
Source: JCI Insight. 2023 Apr 24;8(8):e166811. doi: 10.1172/jci.insight.166811 (PMC10243738; doi:10.1172/jci.insight.166811)
Supplement: Supplemental data [file jciinsight-8-166811-s114.pdf]

**Table S1: GenBank accession numbers of SOSV soluble antigens and SOSV mAbs**		
**GenBank submission name**	**Protein name in paper and description**	**Accession #**
SOSV_HN-ecto	HN_{ecto}; soluble HN protein residues 75-583 with N-terminal 6x His-Tag	OQ384117
SOSV_HN-head	HN_{head}; soluble HN protein residues 125-583 with N-terminal 6x His-Tag	OQ384118
SOSV_postfusion_F_protein	postF-tHS; soluble postfusion F protein. Includes residues 20-481 of SOSV F protein sequence and His- and Strep-tags	OQ384119
SOSV_prefusion_F_protein	preF-tHS; soluble pre-fusion stabilized F protein. Includes residues 20-481 of SOSV F protein sequence with modifications at furin cleavage site and addition of a stabilizing disulfide-bond as well as His- and Strep-tags	OQ384120
SOSV-2_heavy	Heavy chain sequence; F-specific mAb	OQ384121
SOSV-2_light	Light chain sequence; F-specific mAb	OQ384122
SOSV-5_heavy	Heavy chain sequence; F-specific mAb	OQ384123
SOSV-5_light	Light chain sequence; F-specific mAb	OQ384124
SOSV-10_heavy	Heavy chain sequence; F-specific mAb	OQ384125
SOSV-10_light	Light chain sequence; F-specific mAb	OQ384126
SOSV-13_heavy	Heavy chain sequence; HN-specific mAb	OQ384127
SOSV-13_light	Light chain sequence; HN-specific mAb	OQ384128
SOSV-19_heavy	Heavy chain sequence; HN-specific mAb	OQ384129
SOSV-19_light	Light chain sequence; HN-specific mAb	OQ384130
SOSV-21_heavy	Heavy chain sequence; F-specific mAb	OQ384131
SOSV-21_light	Light chain sequence; F-specific mAb	OQ384132
SOSV-23_heavy	Heavy chain sequence; F-specific mAb	OQ384133
SOSV-23_light	Light chain sequence; F-specific mAb	OQ384134
SOSV-24_heavy	Heavy chain sequence; HN-specific mAb	OQ384135
SOSV-24_light	Light chain sequence; HN-specific mAb	OQ384136
SOSV-29_heavy	Heavy chain sequence; HN-specific mAb	OQ384137
SOSV-29_light	Light chain sequence; HN-specific mAb	OQ384138
SOSV-32_heavy	Heavy chain sequence; F-specific mAb	OQ384139
SOSV-32_light	Light chain sequence; F-specific mAb	OQ384140
SOSV-35_heavy	Heavy chain sequence; F-specific mAb	OQ384141
SOSV-35_light	Light chain sequence; F-specific mAb	OQ384142
SOSV-38_heavy	Heavy chain sequence; F-specific mAb	OQ384143
SOSV-38_light	Light chain sequence; F-specific mAb	OQ384144
SOSV-39_heavy	Heavy chain sequence; F-specific mAb	OQ384145
SOSV-39_light	Light chain sequence; F-specific mAb	OQ384146
SOSV-44_heavy	Heavy chain sequence; F-specific mAb	OQ384147

SOSV-44_light	Light chain sequence; F-specific mAb	OQ384148
SOSV-53_heavy	Heavy chain sequence; F-specific mAb	OQ384149
SOSV-53_light	Light chain sequence; F-specific mAb	OQ384150
SOSV-59_heavy	Heavy chain sequence; F-specific mAb	OQ384151
SOSV-59_light	Light chain sequence; F-specific mAb	OQ384152
SOSV-64_heavy	Heavy chain sequence; F-specific mAb	OQ384153
SOSV-64_light	Light chain sequence; F-specific mAb	OQ384154
SOSV-66_heavy	Heavy chain sequence; F-specific mAb	OQ384155
SOSV-66_light	Light chain sequence; F-specific mAb	OQ384156
SOSV-68_heavy	Heavy chain sequence; F-specific mAb	OQ384157
SOSV-68_light	Light chain sequence; F-specific mAb	OQ384158
SOSV-73_heavy	Heavy chain sequence; F-specific mAb	OQ384159
SOSV-73_light	Light chain sequence; F-specific mAb	OQ384160
SOSV-77_heavy	Heavy chain sequence; F-specific mAb	OQ384161
SOSV-77_light	Light chain sequence; F-specific mAb	OQ384162
SOSV-83_heavy	Heavy chain sequence; HN-specific mAb	OQ384163
SOSV-83_light	Light chain sequence; HN-specific mAb	OQ384164
SOSV-84_heavy	Heavy chain sequence; HN-specific mAb	OQ384165
SOSV-84_light	Light chain sequence; HN-specific mAb	OQ384166
SOSV-85_heavy	Heavy chain sequence; F-specific mAb	OQ384167
SOSV-85_light	Light chain sequence; F-specific mAb	OQ384168

All four soluble SOSV antigen protein sequences (HN_{ecto}, HN_{head}, postF-tHS, and preF-tHS) were uploaded to GenBank (OQ384117 through OQ384120). All the V-gene sequence data for the heavy and light chain of the entire panel of 24 anti-SOSV mAbs was also submitted to GenBank (OQ384121 through OQ384168). These sequences were used to produce the recombinant SOSV mAbs used in this body of work.
